# Characterization of *Pseudomonas* Species Isolated from the Rhizosphere of Plants Grown in Serozem Soil, Semi-Arid Region of Uzbekistan

**DOI:** 10.1100/tsw.2005.64

**Published:** 2005-07-08

**Authors:** Dilfuza Egamberdiyeva

**Affiliations:** Center of Agroecology, University of Agriculture, Tashkent 700128, Uzbekistan

**Keywords:** Pseudomonas, enzyme, auxin (IAA), wheat, corn, tomato, plant growth–promoting rhizobacteria (PGPR)

## Abstract

Collections of native *Pseudomonas* spp. are kept at the NCAM of Uzbekistan. Some of those organisms were isolated from the rhizosphere of cotton, wheat, corn, melon, alfalfa, and tomato grown in field locations within a semi-arid region of Uzbekistan. Strains used for this study were Pseudomonas alcaligenes, *P. aurantiaca*, *P. aureofaciens*, *P. denitrificans*, *P. mendocina*, *P. rathonis*, and *P. stutzeri*. Some of the pseudomonads have been characterized in this report. These strains produced enzymes, phytohormone auxin (IAA), and were antagonist against plant pathogenic fungi in *in vitro* experiments. Most of the strains were salt tolerant and temperature resistant. Some of the *Pseudomonas spp*. isolated in this study have been found to increase the growth of wheat, corn, and tomato in pot experiments.

## INTRODUCTION

Studies in microbial diversity are important in order to understand the role of microorganisms in the ecology of soil and other ecosystems[1,2,3]. The genus *Pseudomonas* encompasses arguably the most diverse and ecologically significant group of bacteria on the planet and is found in large numbers in all of the major natural environments and also in associations with plants. This universal distribution suggests a remarkable degree of physiological and genetic adaptability[4].

Investigating the ecology of bacteria associated with plant roots is important to develop an understanding regarding the impact that new agricultural technologies will have on soil ecology, nutrient transformations, and plant succession[5]. *Pseudomonas* spp. are a major component of the microbial flora, which live in close association with various types of agricultural crops. Their association with plant materials has been related both to their antagonistic activities towards pathogens and to their ability to colonize and produce plant growth–promoting compounds within the rhizosphere[6,7]. The *Pseudomonas*

strains have been shown to increase plant growth and nutrient uptake of maize, wheat, and legumes in different soils and temperatures[8,9,10].

There are some reports that describe the physiological characterization of some *Pseudomonas* spp. isolated from different climatic regions[11,12,13]. However, knowledge about the characterization and possible function of *Pseudomonas* spp. in environmentally stressed conditions, such as nutrient-deficient soils within hot dry climates, is limited. In arid regions within Uzbekistan, for example, evaporation of the Aral Sea and the inappropriate application of chemical fertilizers and pesticides has resulted in pollution and salinization of soils and water courses. In the present study, *Pseudomonas* spp. isolated from a semi-arid region of Uzbekistan were examined and their potential role within that ecosystem discussed.

## MATERIALS AND METHODS

### Study Sites and Soil Characterization

Bacterial strains of *Pseudomonas* spp. were taken from the National Collection of Agricultural Microorganisms (NCAM) Uzbekistan. The bacterial strain *P. alcaligenes* PsA15 was isolated from the rhizosphere of melon, *P. aurantiaca* PsAr56 from corn, *P. aureofaciens* PsAf5 from cotton, *P. denitrificans* PsD6 from tomato, *P. mendocina* PsM13 from wheat, *P. rathonis* PsR20 from alfalfa, and *P. stutzeri* PsS23 from wheat grown in calcareous serozem soil, field site of semi-arid region. (Serozem soil conditions are shown in Table 1.)

**TABLE 1**
**Soil Chemical Properties and Soil Particle Distribution at 0- to 30-cm Soil Layer**

| Type | C | N | P | K | pH | Soil Particle Size, mm | | |
|---|---|---|---|---|---|---|---|---|
| | mg · (100 g)$^{-1}$ | | | | | 2–0.2% | 0.2–0.02% | <0.02% |
| Serozem | 200 | 0.6 | 3.0 | 12.0 | 7.5 | 2.2 | 54.5 | 43.3 |

### Morphological, Physiological, and Biochemical Tests

Production of oxidase was determined as described by Cappuccino and Sherman[14]. The catalase production was determined by adding the $H_2O_2$ (3% vol/vol) to a bacterial culture and the presence of catalase indicated by bubbles of free oxygen gas ($O_2$)[14]. Formation of fluorescent pigment was observed on King B medium[15]. Production of pyocyanin was read after 3 d at 28$^{o}$C with King's A medium[15]. Acid production from carbohydrates was tested in peptone water broths containing 1% carbohydrate and Andrade's indicator[16]. Colony morphologies were examined after 24-, 48-, and 72-h growth on glycerol peptone agar (GPA) at 28$^{o}$C. Cell morphologies were examined with phase contrast microscopy and after staining with methylene blue. The oxidation and fermentation of glucose was performed according to the method of Hugh and Leifson[17].

Arginine dihydrolase was performed according to the method of Thornley[18]. Hydrolysis of starch, casein, and lecithin was determined as described by Smibert and Krieg[19]. The plates were incubated at 28$^{o}$C and zones of starch cleaning recorded after 5 d. Hydrolysis of Tween 20 and 60 was determined on modified sierra agar, containing 10 g of peptone, 3 g of meat extract, 5 g of NaCl, 0.2 g of Fe-citrate, 0.1 g of $CaCl_2.H_2O$, and 15 g of agar in 1 l of distilled water. Ten ml of sterile Tween 20 and 60 and 50 ml of 0.067% (w/v) Victoria Blue B solution were added to the medium after autoclaving. Hydrolysis of Tween was recorded as white precipitation around the colonies. The method of Jayasankar and Graham[20] was used to determine the presence of pectinase and cellulase. Gelatinase activity was detected by replacing agar with gelatine as a gelling agent in GPA dispensed in stab culture tubes and by looking for liquefaction after 7 d incubation at 20$^o$C. Indol production from tryptophan was tested using the method of Clarke and Cowan[21]. Production of $H_2S$ was read after 7 d incubation at 28$^o$C in triple iron salts agar[16]. Acetilmethylcarbinol production was carried out according to the method of Clarke and Cowan[21]. Urease activity (Christiansen's method), citrate utilization, lipase production, hydrolysis of tryptophan, and nitrate reductase were carried out as described in Cowan[16]. Growth at different temperatures was observed in MPA medium after incubation at +4 (for 10 d) and +50$^o$C (for 5 d). Salt tolerance was determined in GPA medium containing NaCl at 7% w/v. Auxin production was tested using Salkowsky's reagent[22]. Plant pathogenic fungi (*Fusarium culmorum*, *Verticillum loteritum*) and soil fungi (*Aspergillus flavus, A. insultus, A. ustus, Penicillum purpurogenum, P. sopii,* and *Trichoderma lignorum*) were used as indicator strains for the observation of potentially antagonistic pseudomonads using simple plate assay methods. This involved preincubation of the putative antagonist on yeast malt agar at 28$^o$C for 2 d. Plates were then seeded with individual fungal strains and incubated for a further 5 d in a sealed container. Zones of inhibition were then recorded.

The study of the effect of isolated strains on plant growth of wheat, corn, and tomato was carried out in pot experiments using a nutrient-poor serozem soil taken from field site. The inoculation treatments were set up in a randomized design with six replicates for each strain tested. The day before sowing, pots were filled with 350 g of soil. Four seeds of wheat and tomato and three seeds of corn were sown per pot. After germination, plants were thinned to two per pot. Wheat, tomato, and corn seedlings were inoculated *in situ* with 1 ml of the bacterial suspension (ca. $10^6$ cfu/ml) that was incubated on a rotary shaker (120 rpm; 26°C) for 3 d using glycerine-peptone medium. Uninoculated plants were considered as control plants. Plants were grown in pots for 4 weeks under greenhouse conditions with a temperature of 32–34°C during the day and 18–22°C at night. The soil was moistened with sterile water and maintained at 60% of its moisture holding capacity (MHC). Four weeks after germination, the shoot and root length were measured, separated, and dried at 105°C, before determining their respective dry weights.

The data were analyzed with an ANOVA and Student-Newman-Keuls test for testing the significant differences ($p < 0.05$) of main effects.

## RESULTS

### Characterization of Bacteria Isolated

Biochemical characterization of all the pseudomonads isolated is shown in Table 2. All bacterial strains studied were oxidase positive and reduced nitrate to nitrite. Three strains *Pseudomonas denitrificans* PsD6, *P. rathonis* PsR 20, and *P. stutzeri* PsS23 produced arginine dihydrolase. Only one strain, *P. rathonis* PsR20, was catalase negative. None of the strains produced fluorescein or pyocyanin. Only one strain (*P. denitrificans* PsD6) had lecitinase activity and two strains (*P. rathonis* PsR20 and *P. mendocina* PsM13) had cellulase activity. A test for indol production was negative for all strains and urease was only produced by *P. rathonis* PSR20.

Most strains grew at 10$^o$C, while *P. alcaligenes* PsA15, *P. stutzeri* PsS23, *P. aurantiaca* PsAr56, and *P. rathonis* PsR20 grew at 50$^o$C. All strains except PsD6 were also tolerant to 7% NaCl (Table 2).

The patterns of acid production from carbohydrates produced by the *Pseudomonas* isolates are shown in Table 3. Four strains produced acid from glucose and three strains produced acid from mannitol. None of the strains examined produced acids from galactose, arabinose, and xylose.

Hydrolytic reactions of the test strains showed that *P. alcaligenes* PsA15 and *P. rathonis* PsR20 hydrolyzed starch, Tween 20, and Tween 60. Starch was hydrolyzed by five of the six of strains tested (see Table 4).

**TABLE 2**
**Biochemical Characteristics of *Pseudomonas* spp.**

| Property Tested | Bacterial Strains | | | | | | |
|---|---|---|---|---|---|---|---|
| | PsA15 | PsAf5 | PsAr56 | PsD6 | PsM13 | PsR20 | PsS23 |
| Casein hydrolysis | – | – | – | – | – | – | – |
| Gelatine liquefaction | + | – | – | – | – | – | – |
| Citrate utilization | + | + | – | + | + | + | + |
| Arginine dihydrolase | – | – | – | + | – | + | + |
| Acetilmethilcarbinol | – | – | – | – | – | – | – |
| Metabolism O | + | + | + | + | + | + | + |
| F | – | – | – | – | – | – | – |
| Growth at 4°C | – | – | – | – | – | – | – |
| 50°C | + | – | + | – | – | + | + |
| Growth at 7% NaCl | + | + | + | – | + | + | + |
| $H_2S$ | + | – | – | – | + | + | + |
| Oxidase | + | + | + | + | + | + | + |
| Catalase | + | + | + | + | + | – | + |
| Lipase | + | – | – | – | – | + | + |
| Tryptophanase | – | – | – | – | – | – | – |
| Nitrate reductase | + | + | + | + | + | + | + |
| Amylase | + | – | – | – | – | + | + |
| Collagenase | + | – | – | – | – | + | – |
| Pectinase | + | – | + | – | – | – | + |
| Lecithinase | – | – | – | + | – | – | – |
| Cellulase | – | – | – | – | + | + | – |
| Urease | – | – | – | – | – | + | – |

**TABLE 3**
**Carbohydrate Fermentation Pattern of *Pseudomonas* spp.**

| Acid Source | PsA15 | PsAf5 | PsAr56 | PsD6 | PsM13 | PsR20 | PsS23 |
|---|---|---|---|---|---|---|---|
| Arabinose | – | – | – | – | – | – | – |
| Xylose | – | – | – | – | – | – | – |
| Glucose | + | – | – | – | + | + | + |
| Galactose | – | – | – | – | – | – | – |
| Saccharose | + | – | – | – | – | – | – |
| Lactose | – | – | – | – | – | – | + |
| Maltose | – | – | – | – | – | – | + |
| Glycerol | – | – | – | – | + | – | + |
| Mannitol | – | – | – | – | + | + | + |

The microbial production of antifungal metabolites by bacteria provides an important source of useful molecules within any ecosystem. All six pseudomonads isolated were antagonistic to one or more of the soil fungi *A. flavus, A. insultus, A. ustus, Penicillum purpurogenum, P. sopii,* and *T. lignorum.* The growth of most of the above fungi was strongly inhibited by *Pseudomonas aureofaciens* PsAf5 (Table 5). Only two strains (*P. alcaligenes* PsA15 and *P. denitrificans* PsD6) were antagonist towards the plant pathogenic fungus *Vertilcillum loteritum* (data not shown).

**TABLE 4**
**Activity of *Pseudomonas* spp. to Hydrolyze Starch and Tween *in situ***

| Bacterial Strains | Starch (cm) | Tween 20 (mm) | Tween 60 (mm) |
|---|---|---|---|
| *P. alcaligenes* PsA15 | 1.5* | 2.2 | 2 |
| *P. aureofaciens* PsAf5 | 6 | — | — |
| *P. denitrificans* PsD6 | — | 1 | — |
| *P. rathonis* PsR20 | 2.5 | 3 | 2 |
| *P. stutzeri* PsS23 | 3.8 | — | — |
| *P. stutzeri* PsS25 | 3.5 | 3 | — |

* Diameter of hydrolytic zone in cm.

**TABLE 5**
**Antagonistic Activity of *Pseudomonas* spp. Against Nonpathogenic Fungi**

| Bacterial Strains | *Aspergillus flavus* | *Aspergillus insultus* | *Aspergillus ustus* | *Penicillum purpurogenum* | *Penicillium sopii* | *Trichoderma lignorum* |
|---|---|---|---|---|---|---|
| PsA15 | — | 2* | — | 3* | 2* | 3* |
| PsAf5 | 3* | 3* | 2* | — | 2* | — |
| PsD6 | — | 2* | — | — | 2* | 2* |
| PsM13 | 3* | — | — | 2* | — | 2* |
| PsR20 | — | 2* | 4* | — | — | 3* |
| PsS23 | — | 2* | — | — | — | 2* |

* Inhibition zone in mm.

A rapid screening of auxin production showed that two bacterial isolates produced auxins. These were *P. alcaligenes* PsA15, which produced 3.2 µg IAA 100 $ml^{-1}$ filtrate, and *P. denitrificans* PsD6, which produced 3.0 µg IAA 100 $ml^{-1}$ filtrate (data not shown).

In terms of improved plant growth, some of the bacterial strains examined increased the growth (expressed as shoot and root lengths) of wheat, corn, and tomato in a nitrogen-deficient soil. Independent of the origin from where bacterial strains have been isolated, *P. denitrificans* PsD6, *P. rathonis* PsR20, and *P. aureofaciens* PsAf5 increased the shoot and root length of wheat and corn (from 30–45%) compared to the control plants (Figs. 1 and 2). The inoculation of tomato also resulted in increase of shoot and root length of plant grown in serozem soil (Fig. 3)

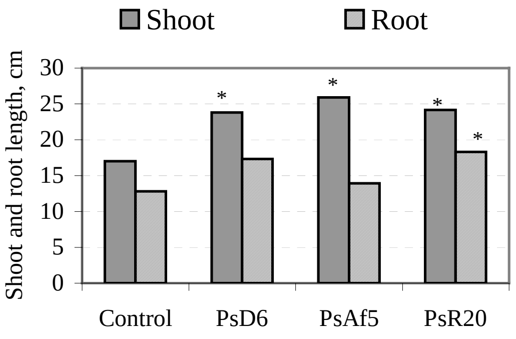

**FIGURE 1**. Effect of *Pseudomonas* spp. on shoot and root growth of wheat, *P. denitrificans* PsD6, *P. aureofaciens* PsAf5, and *P. rathonis* PsR20. *Significant differences, $p < 0.05$.

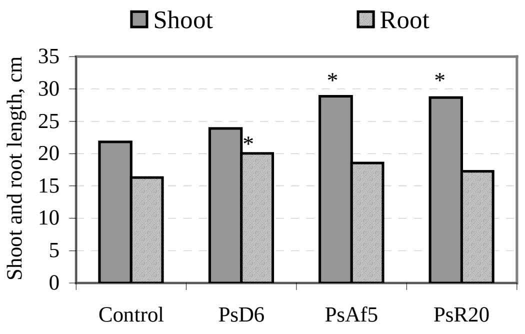

**FIGURE 2**. Effect of *Pseudomonas* spp. on shoot and root growth of maize, *P. denitrificans* PsD6, *P. aureofaciens* PsAf5, and *P. rathonis* PsR20. *Significant differences, $p < 0.05$.

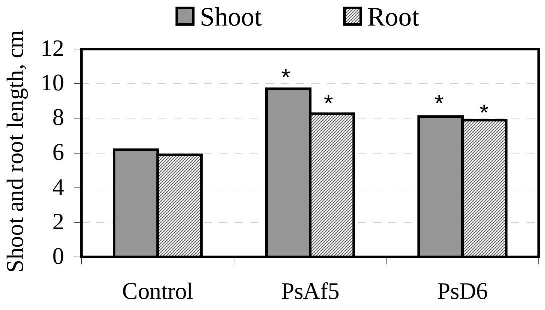

**FIGURE 3**. Effect of *Pseudomonas* spp. on shoot and root length of tomato, *P. denitrificans* PsD6, and *P. aureofaciens* PsAf5. *Significant differences, $p < 0.05$.

## DISCUSSION

Pseudomonads are well suited to the rhizosphere because they are able to use a wide variety of carbon sources as nutrients. Like many rhizosphere bacteria, they play a role in nutrient cycling, can be plant growth–promoting rhizobacteria (PGPR), and may act as biological control agents (BCA). They exhibit a wide range of metabolic activities and are able to utilize a wide range of low molecular mass organic compounds, and some more complex compounds, as carbon and energy sources[23].

In early studies, Stainer et al.[24] described the remarkable capacity of *Pseudomonas* strains to degrade a wide range of substrates including aromatic compounds, halogenated derivatives, and growth characteristics of 267 strains on 146 different organic compounds, plus a wide range of associated tests. In this present work, we describe the biochemical and physiological characterization of *Pseudomonas* strains isolated from alfalfa, corn, cotton, melon, tomato, and wheat grown in a calcareous serozem soil, in a semi-arid region of Uzbekistan.

Our *Pseudomonas* strains produce different biologically active compounds. There are many reports that note the production of biologically active compounds including different enzymes, phytohormones (such as auxins), and also siderophores by rhizosphere bacteria[9,10,25,26]. The bacterial strains that produce different hydrolytic enzymes such as protease, lipase, and pectinase were effected antagonistically to soil fungi. Abd Rahman et al.[27], Caballero et al.[28], and Hutberg et al [29] reported that among studied pseudomonads, the *P. aureginosa* produced several proteases that have implicated in its pathogenicity. Neiendam Nielson and Sorensen[30] demonstrated that isolates of *P. fluorescens* antagonistic to *Rhizoctonia solani* and *Pythium ultimum*, produced lytic enzymes.

Organic substances capable of regulating plant growth produced either endogenously or applied exogenously are called plant growth regulators. They regulate growth by affecting physiological and morphological processes at very low concentrations[31]. Microbial IAA has been implicated in the stimulation of growth of plants[22]. A diverse group (including pseudomonads) has been found to synthesize IAA[32]. IAA influences root length due the hormonal effect. In this study, two bacterial strains produced considerable amounts of IAA, which are comparable with earlier studies on various bacteria including *Pseudomonas*[33,34]. Dey et al.[35] reported that *Pseudomonas* spp. strains isolated from peanut produced IAA and inhibited a fungal pathogen including *A. niger*. Seed bacterization of these isolates increased the root length and plant height in pots significantly over the control. *Pseudomonas* spp. strains isolated from the rhizosphere of maize, wheat, rice, and soybean produced IAA, utilized starch, and were positive for lipase activity. Monosaccharides (glucose, mannitol fructose, and sorbitol) were utilized by all the bacterial isolates[34].

The ability of the seven isolates of *Pseudomonas* spp. from this study to tolerate high temperatures and salt concentrations thus uncommon among terrestrial pseudomonads thus confer on the isolates a potential competitive advantage to survive in arid and saline regions of the world.

Coupled with their ability to survive in warm saline climates, bacteria *P. denitrificans* PsD6, *P. aureofaciens* PsAf5, and *P. rathonis* PsR20 were shown, under glasshouse experimental conditions, to contribute towards the increased plant growth of wheat, corn, and tomato in soils; while nontreated control plants by comparison also performed poorly under such conditions.

De Freitas and Germida[36] also demonstrated that in a low-fertility soil, pseudomonads significantly enhanced early plant growth of winter wheat. According to Lazarovitz and Nowak[37], rhizosphere bacterization of crop plants only marginally increased yields when tested under ideal climatic situations and greatest benefits in terms of improved plant health and yield were obtained when crops encountered environmental stress (e.g., elevated temperatures) for prolonged periods. Some successful examples of inoculation with PGPR have been achieved both in laboratory and field trials. For example, strains of *P. putida* and *P. fluorescens* could increase root and shoot elongation in canola, lettuce, and tomato[38]. *P. aureofaciens* strain AB254 increased seedling emergence compared with that of untreated seed of corn[39].

In conclusion, based on this study, we have shown that *Pseudomonas* spp. isolated from the semi-arid saline serozem soil of Uzbekistan have the potential to produce different biologically active compounds and may have the ability to survive in ecologically stressed conditions including hot summer temperatures and saline and nitrogen-deficient soils. In addition, some strains have the ability to increase plant growth and stimulate early growth. Further work is required to trial an appropriate mix of strains in order to develop pilot "biofertilizers" for use in the Uzbek agricultural system. Such biofertilizers may offer the potential of an alternative, improved, and more environmentally friendly regime than does the current status quo in Uzbek agricultural practice.

## ACKNOWLEDGMENT

The author would like to thank Dr. Peter Green (NCIMB UK) for supervision and critical comments on the manuscript.

## BIOSKETCH

**Dilfuza Egamberdiyeva** is a head of the Soil Microbiology and Plant–Microbe Interactions Laboratory and curator of the National Collection of Agricultural Microorganisms (NCAM) at the University of Agriculture, Uzbekistan. She holds degrees in agriculture from the Humboldt University of Berlin, Germany. Her current research interests lie in microbial diversity and community analysis, rhizobacteria, plant microbe interactions research, biological control, and development of bacterial fertilizers for agricultural practices.
